# The Essentials of Histology

**Published:** 1886-05

**Authors:** 


THE ESSENTIALS OF HISTOLOGY, DESCRIPTIVE AND PRACTICAL, FOR THE USE OF STUDENTS. *By* E. A. SCHAFER, F. R. S. *Jodrell Professor of Physiology in University College, London; Editor of the Histological Portion of Quain's Anatomy. Philadelphia:* LEA BROTHERS & CO. *1885. Chicago:* W. T. KEENER.

This book is intended as a working manual for the laboratory as well as a text-book on histology.

The methods of the study of the tissues, though not in every case the best possible, are all good, and above all practical in laboratory teaching.

The histology is founded upon the second volume of Quain's anatomy, written by the same author. Those who are acquainted with Quain will expect a high grade of performance here, nor will such expectation be disappointed. The author seems to have recognized the fact that in a work of this kind it is his duty to give the state of the science as it is received by the best authorities, and not to air his own notions. This aim he has adhered to, admirably. A significant difference between the descriptions of the white blood-corpuscles in this volume and that in Quain, will illustrate. In Quain, Klein's figure of the network in the white corpuscles is reproduced and endorsed. In the present volume the figure is absent, and all he says is that "articular structure is described by some histologists." He seems doubtful of the facts. This conservatism as to theories does not prevent his accepting new facts promptly, as may be seen in his description of a villus.

But no human work is perfect. He has not seen the eminence in the centre of the red blood-corpuscles, called by some a nucleus, though he mentions it, probably because he will not use the proper illuminating apparatus, very likely regarding all such things, in common with so many other histologists, as playthings.

He gives the number of the red corpuscles in a cubic millimeter of blood as from four to five millions, although the best authorities say the number is at least a million more.

Perhaps the most careless part of the book is on the kidney. In a table of the course and position of the tubules with the character of their epithelium he says the "epithelium of the convoluted tubule is cubical, fibrillated, ciliated," etc. In the text he says that "in many animals" these cells "have been shown to be ciliated." He does not state, however, that the cilia have been found in the *neck of the tube only*, where it passes out from the malpighian capsule and that it may be the remains of a fœtal structure, and is not always present in these animals. The cilia are found in the tubes in the lowest animals only, such as birds and reptiles that have solid or viscid urine. They have never been demonstrated in any part of the kidney in man and most of the higher mammals. It would seem to be a matter of no such extraordinary difficulty to find them with the modern methods, if they really are present, when they can be shown in the central canal of the spinal cord with such comparative ease.

One who was critical might find other places not faultless, but occasions for censure are rare. On the whole, the book is excellent, and supplies a want, there being no histology in the market adapted to the wants of the student except, perhaps, Quain; but for most students Quain is out of reach. L. C.

